# A Year of Progress

**Published:** 1909-12-25

**Authors:** 


### IN THE HOSPITAL WORLD.

### A YEAR OF PROGRESS.

No one who is familiar with the actual facts will dispute that everywhere throughout the English-speaking world hospital questions have steadily progressed during 1909. The same to a large extent may be said of Germany and of some other Continental nations in regard to certain aspects of the hospital, especially its structure and hygienic completeness. In North America there can be no question that the development of the American Hospital Association has had a material influence for good not only upon voluntary hospitals but upon all hospitals which properly bear that name in the United States. We believe that the reorganisation upon a wider and firmer basis of the British Hospitals Association, with its annual congresses, and the spread of matured opinion upon many important hospital questions which must follow such meetings, will be attended with the best results. It has been our privilege recently to make a long and exhaustive tour of inspection of most of the principal voluntary hospitals and poor-law infirmaries situated in England and Wales. These inspections are not yet completed, but they are so numerous already as to enable us to declare that, speaking generally, the voluntary hospital system throughout England is sound, and that, when less than ten existing buildings have been scrapped, and new hospitals have been built, and when some dozen others have reconsidered their existing rules and modernised and brought them up to date, the voluntary hospitals throughout England will be found to typify the best form of guest-house for the sick which the world has ever possessed. The administration and habitat of our voluntary hospitals being thus shown to be sound and good, it may be well to turn to the bedrock on which they depend—namely, finance.

### The Voluntary System is Financially Sound.

If public opinion were guided by the opinions of certain young men in a hurry, the English people, and indeed the British people too, might conclude that the voluntary hospital system had fallen into decay; and that the blessed work of relieving the sick and injured was emphatically not a work for private charity at all, but that it belonged to the municipality or the State. In order to justify a wanton statement of this kind, which has no solid foundation to rest upon whatever, the same youthful and hurried critics naturally maintain, as they have to maintain, that as a fact there has been a gradual and constant falling off in the support which has been given to charities—charities, be it understood, of all kinds, not merely hospitals, for of course such a critic must deal with immense figures without probably any real knowledge or apprehension of what they amount to in fact. We have been at some pains during the last few years to get out the figures, which will be found in successive volumes of "Burdett's Hospitals and Charities." There will be found the returns of the income and expenditure of upwards of two thousand charities of all kinds, including hospitals, dispensaries, convalescent homes, missions, orphanages, homes and charities, institutions for the blind, for the deaf and dumb, for chronic and incurable cases, together with administrative and collective societies. These two thousand charities have collectively an income which exceeds twelve millions per annum, and, so far from the voluntary support of these charities falling off, the latest returns show that they have an excess of income over expenditure which, on an average, considerably exceeded one million sterling in each of the years 1905, 1906, and 1907. We may state for the information of the sceptics, young and old, that we have taken the trouble to go back to 1896, when the total income of 1,867 charities of all kinds was only a little over eight millions, with the result that the total income exceeded the expenditure at that date by upwards of half a million sterling. So much for the principal British charities.

### The Voluntary Hospital System is Sound.

When the critic, being a hospital man, deals with hospital finance, he ought to show more and better knowledge of his subject. Unfortunately he gets deeper and deeper into the slough of irrelevant inaccuracies. We have met this type of critic in various forms, and have answered him in various ways for quite thirty years past, but the type is always with us, and we should welcome his presence were it not for the fact that recently a few of the younger men, whose manhood ought to give them more pluck and energy and knowledge, have been tempted, apparently, to ease the yoke from their shoulders, as upholders of the voluntary hospital system, by crying it down in favour of the municipality or the State. One of them has recently declared in public that the chief cause of the decay of the voluntary system, and its failure to finance the hospitals of this country, was to be found in this feeling on the part of many of the charitable that the work which the hospitals were doing was a work which, so far as voluntary hospitals were concerned, had had its day. Had its day, forsooth! Why, **the audited figures nail this falsehood to the table, and prove that the day of the voluntary system in this country is only at its dawn.** Such palpable errors become ridiculous when judged by the continuous growth which has taken place in the amount of money which has been freely given for the support of voluntary hospitals every year, for more years than the particular speaker has probably lived on the face of the earth. Let us take the hospital figures. We find that, whereas in 1896 716 hospitals expended altogether upwards of two and a quarter millions, they received upwards of two and a half millions, and had a surplus of a quarter of a million when everything was paid. Ten years later, in 1905, the number of the voluntary hospitals, for which figures were available, had increased to 794, and their total expenditure to £3,350,000, whilst their surplus of income over expenditure had more than doubled, for it amounted to no less a sum than £542,500. We find, too, that the voluntary hospitals have increased in number every year since, and that each year there has been a splendid surplus of total income over total expenditure. Thus, in 1906 796 hospitals had a surplus revenue of £351,637, and in 1907 803 voluntary hospitals had a surplus revenue of £482,649. We have no space here to go into the details of the figures, which will be found in Burdett's "Hospitals and Charities" for 1908 and 1909, whilst the latest figures, those for 1908, will be found in the volume of this book for 1910 now in the press. It is further remarkable that every year from 1905 onwards the ordinary income has increased, the special donations to building and other funds have maintained a splendid average of considerably over half a million sterling each year, and that the largest sum received in the period from legacies was during the last year, 1907. We hope, therefore, that no one connected with a reputable voluntary hospital will ever again associate his name with a statement so false as that which has recently been published, in which it is stated that those engaged in financing the voluntary hospitals were bolstering up a system which was bound to come to an end. It is true, of course, that Socialism is in the air, and that the Socialist, who unfortunately has not a knowledge of the relative efficiency of voluntary as opposed to State hospitals all the world over, has set his soul upon receiving everything from the State or the municipality, and regards this evidence of degeneracy in a nation or people as the highest goal which he and his party should strive to reach. We expect better things, however, and the exhibition of knowledge and cheerfulness and courage from every man and woman who has devoted his or her life to the work of making more and more efficient the voluntary hospital, or hospitals, with which they may be connected. **Now it is untrue that the voluntary hospital system, when put to the proof, shows any evidence "of the gradual and constant falling off in the support of the public."** On the contrary, the financial position and published accounts of the voluntary charities of this country afford no such evidence. Indeed, we have proved by figures, which are taken from the audited reports of two thousand charities of all classes, including eight hundred voluntary hospitals, that the exact contrary is the case. The figures indicate, despite the enormous growth in the cost of maintaining the voluntary hospitals, that the contributions of the public every year have amounted to a total which far exceeds the total expenditure required for the efficient maintenance, upkeep, and rebuilding of our voluntary hospitals. Further than this, **twenty millions sterling at least in the last 20 years have been provided for the sick poor of these islands by free gifts, to enable the older and many of the larger, more important, and popular of our voluntary hospitals to be entirely reconstructed or rebuilt.**

The King's Hospital Fund.

The late Queen Victoria was the mother of her people, and her diamond jubilee, as events have proved, has been fittingly and nobly commemorated by the foundation of King Edward's Hospital Fund which is now incorporated by statute. That Fund has become a great central board, which is exercising full and wise control over the metropolitan hospitals, their government and finances, to the great advantage of everyone concerned. This Fund was founded twelve years ago to raise and distribute a maximum of £150,000 a year amongst the voluntary hospitals of London. In 1909 it fully attained its object, and will have a surplus revenue in addition. It is remarkable, too, that it should have invested funds exceeding £1,600,000, and that the revenue from small subscriptions of 1s. a year and upwards, through the League of Mercy, has enabled £21,000 to be given to the hospitals in 1909 from this source alone, whilst the League's revenues show a tendency to increase and extend. For causes which we need not further refer to in this place, the one source of revenue which is not satisfactory is that derived from ordinary subscriptions and donations. As the Prince of Wales said at the annual meeting in December, there is reason to hope that these causes are temporary, and that before long the £50,000 a year which it was the aim of the founders to obtain from this source may be forthcoming. At any rate, it is striking testimony to the vitality of the voluntary system in this country that a new fund, which was met at its birth by opposition and criticism, should have attained success within twelve years.

But the King's Fund has done much more than finance the metropolitan hospitals. It has had a most marked effect for good by restraining ill-considered expenditure upon bricks and mortar, and it has further produced through the publication of accurate statistical tables and returns a saving in the expenditure upon certain voluntary hospitals of the metropolis which exceeds £39,000 per annum. Once again the King's Fund has organised a system of visitation, which enables the Council and its committees to have before them accurate details of the inner working and actual state of every institution which applies for a grant each year. This system of visitation has educated a large number of the leading men of the day, representing all professions and ranks of society, to a knowledge of hospital requirements and hospital management, which has been fruitful in results for good. In the same way and by the same system the League of Mercy is securing identical results in an equal number of hospitals scattered throughout the home and contiguous counties of England. These results and the rapidity and success with which they have been obtained, it is but just to state, have been due in large measure to the example and personal work and interest which the King, the Prince and Princess of Wales, and other members of the Royal Family have displayed in the cause of the voluntary hospitals since the King's Fund was instituted in 1897.

